# POU2F3-positive small cell neuroendocrine carcinoma of the urinary tract: a clinicopathological analysis of five cases

**DOI:** 10.3389/fonc.2026.1849428

**Published:** 2026-06-12

**Authors:** Linxi Chen, Hongsheng Lu, Xuequan Cao, Hualei Gan, Peng Liu

**Affiliations:** 1 Taizhou Central Hospital (Taizhou University Hospital), School of Medicine, Taizhou University, Taizhou, Zhejiang, China; 2 Department of Pathology, Fudan University Shanghai Cancer Center, Shanghai, China

**Keywords:** pathology, POU2F3, small cell carcinoma, SmCC, urinary tract neoplasms

## Abstract

**Objective:**

To investigate the expression of POU2F3 in urinary tract small cell carcinoma (SmCC) and evaluate its diagnostic value.

**Methods:**

A retrospective analysis was performed on five cases of POU2F3-positive urinary tract SmCC diagnosed between January 2023 and November 2025 in the Departments of Pathology at Taizhou Central Hospital and Fudan University Shanghai Cancer Center. Immunohistochemistry and next-generation sequencing (NGS) were performed, and the clinicopathological and molecular characteristics were analyzed in combination with a literature review.

**Results:**

Five patients were included in the study group. Two female patients aged 67 and 62 years had bladder tumors and presented with gross hematuria; both underwent transurethral resection of bladder tumor. Three male patients aged 69–70 years had tumors located in the ureter (n=2) and renal pelvis (n=1). Histologically, all tumors showed small round blue cells with a high nuclear-to-cytoplasmic ratio, finely granular chromatin, inconspicuous nucleoli, frequent mitoses, and focal coagulative necrosis. Immunohistochemistry demonstrated loss of conventional neuroendocrine markers (CgA and Syn). Aberrant p53 expression was observed in all cases, and RB1 loss in four cases. Epithelial markers showed weak or paranuclear dot-like positivity. All five cases showed diffuse POU2F3 expression. Six conventional bladder SmCC cases positive for CgA and Syn were used as controls and were all negative for POU2F3. NGS performed in one case revealed a TP53 missense mutation (p.V225I). During follow-up, distant metastases occurred in three patients.

**Conclusion:**

POU2F3 may serve as a useful marker for identifying urinary tract SmCC with absent or low neuroendocrine marker expression. Its application may improve diagnostic accuracy and facilitate recognition of a potential neuroendocrine-low phenotype.

## Introduction

Small cell carcinoma (SmCC) of the urinary tract is a rare and aggressive neuroendocrine tumor, most commonly involving the bladder, and occasionally seen in the upper urinary tract, including the ureter and renal pelvis (1–3). Clinically, patients often present with nonspecific symptoms such as hematuria, dysuria, or lower abdominal pain. Due to its insidious onset and rapid progression, the disease is frequently diagnosed at an advanced stage and is associated with a poor prognosis (3–5). Histologically, SmCC typically exhibits classic small cell morphology and expresses neuroendocrine markers such as synaptophysin (Syn), chromogranin A (CgA), and INSM1. However, recent studies have shown that a subset of tumors lack conventional neuroendocrine marker expression, suggesting underlying molecular heterogeneity (6).

With advances in the molecular classification of small cell lung cancer (SCLC), POU class 2 homeobox 3 (POU2F3) has been identified as a lineage-defining transcription factor for a neuroendocrine-low subtype, termed SCLC-P (7). As a master regulator of tuft cell differentiation, POU2F3 expression is closely associated with reduced or absent neuroendocrine marker expression. This finding has prompted increasing interest in its role in extrapulmonary small cell carcinomas.

To date, POU2F3-positive SmCC of the urinary tract remains poorly characterized, with limited data available. In this study, we report five cases of POU2F3-positive urinary tract SmCC. By characterizing their clinicopathological features, we highlight the diagnostic value of POU2F3 in recognizing a rare neuroendocrine-low phenotype that might be misclassified in routine practice.

## Methods

### Study population

Five cases of POU2F3-positive urinary tract SmCC diagnosed between January 1, 2023 and November 30, 2025 were retrieved from Taizhou Central Hospital and Fudan University Shanghai Cancer Center. The diagnosis of SmCC was established according to the 2022 WHO Classification of Urinary and Male Genital Tumours (8). The cohort included two transurethral resection specimens and three biopsy specimens. Clinical data, including age, sex, tumor location and size, treatment, and follow-up information, were collected. The study was approved by the Ethics Committee of the Taizhou Central Hospital (Taizhou University Hospital).

### Histology and immunohistochemistry

All specimens were fixed in 10% neutral buffered formalin for 24 hours, embedded in paraffin, and sectioned at 4 μm for hematoxylin-eosin staining. Histological features were evaluated and correlated with immunohistochemical findings.

Immunohistochemistry was performed using the EnVision two-step method on a Roche BenchMark XT automated staining system. Antibodies included CK7 (EP16, ZSGB-BIO), CK20 (Ks20.8, Maixin Biotech), CK5/6 (MX040, Maixin Biotech), cytokeratin (AE1/AE3, Maixin Biotech), GATA3 (EP368, Maixin Biotech), Syn (MX038, Maixin Biotech), CgA (MX018, Maixin Biotech), INSM1 (MRQ-70, ZSGB-BIO), NKX2.2 (EP336, Maixin Biotech), RB1 (G3-245, BD Biosciences), p53 (MX008, Maixin Biotech), p16 (MX007, Maixin Biotech), Ki-67 (MXR002, Maixin Biotech), HER2 (MXR001, Maixin Biotech), and POU2F3 (E5N2D, Cell Signaling Technology). Appropriate positive and negative controls were used. All slides were independently reviewed by two experienced pathologists. Consistent with prior studies, POU2F3 expression was considered positive when nuclear staining was present in more than 10% of tumor cells, to avoid classifying minimal or nonspecific staining as positive (6).

### Molecular analysis

Next-generation sequencing was performed in one case using a targeted capture-based panel. Both DNA and RNA extracted from formalin-fixed paraffin-embedded tissue were analyzed using an Illumina sequencing platform.

## Results

### Clinical findings

Among the five patients, two were female and three were male, with a median age of 67 years (range, 62–70 years). None had a prior history of malignancy. The most common presenting symptoms were gross hematuria and lower abdominal pain. The clinicopathologic features of all five cases were summarized in **Table 1**.

**Table 1:** Clinical characteristics and pathological findings of five POU2F3-positive small cell carcinomas of the urinary tract.

Case	Age	Sex	Site of origin	Key immunohistochemical findings	Tumor composition	Recurrence/metastasis	Follow-up
1	67	Female	Bladder (right lateral wall), 2.0 × 1.7 cm	POU2F3 (diffuse+), CgA (-), Syn (-), INSM1 (-), GATA3 (-), p53 (null), Ki-67 (90%)	Pure SmCC	No	17 months
2	62	Female	Bladder (anterior wall), 2.4 × 3.9 cm	POU2F3 (diffuse+), CgA (-), Syn (focal+), INSM1 (-), GATA3 (-), p53(overexpression), Ki-67 (85%)	Pure SmCC	No	17 months
3	70	Male	Left ureter, 2.5 × 2.0 cm	POU2F3 (diffuse+), CgA (-), Syn (-), INSM1 (-), GATA3 (-), p53 (null), Ki-67 (70%)	Pure SmCC	Synchronous metastasis at diagnosis: left iliac regional lymph nodes; metastatic nodules in the T12 left intervertebral foramen and left perirenal space	Lost to follow-up
4	70	Male	Left ureter, 7.7 × 4.8 cm	POU2F3 (diffuse+), CgA (-), Syn (-), GATA3 (-), INSM1 (rare+), p53 (overexpression), Ki-67 (80%)	Pure SmCC	Synchronous metastasis at diagnosis: retroperitoneal mass along left abdominal aorta; multiple regional retroperitoneal lymph nodes; right diaphragmatic crus and adjacent peritoneum	Lost to follow-up
5	69	Male	Right renal pelvis, 4.5 × 3.2 cm	POU2F3 (diffuse+), CgA (rare+), Syn (rare+), INSM1 (rare+), GATA3 (-), p53 (cytoplasmic), Ki-67 (80%)	SmCC with focal high-grade urothelial carcinoma component	Synchronous metastasis at diagnosis: multiple regional retroperitoneal lymph nodes; liver metastasis	Lost to follow-up

### Imaging findings

Preoperative imaging revealed solitary masses ranging from 2.5 cm to 7.7 × 4.8 cm. PET/CT showed a bladder mass in case 1, a suspicious lesion in the distal ureter in case 3, and a large retroperitoneal mass involving the ureter in case 4.

### Pathological findings

Grossly, tumors appeared as gray-white to gray-brown tissue fragments. Microscopically, tumor cells were arranged in sheets or nests, composed of small to oval cells with scant cytoplasm and high nuclear-to-cytoplasmic ratio. Chromatin was finely granular, nucleoli were inconspicuous, and mitotic figures were frequent. Coagulative necrosis and nuclear molding were commonly observed. Depth of invasion varied among cases, although assessment was limited in biopsy specimens.

### Immunohistochemical findings (Figure A–P)

**Figure 1:**
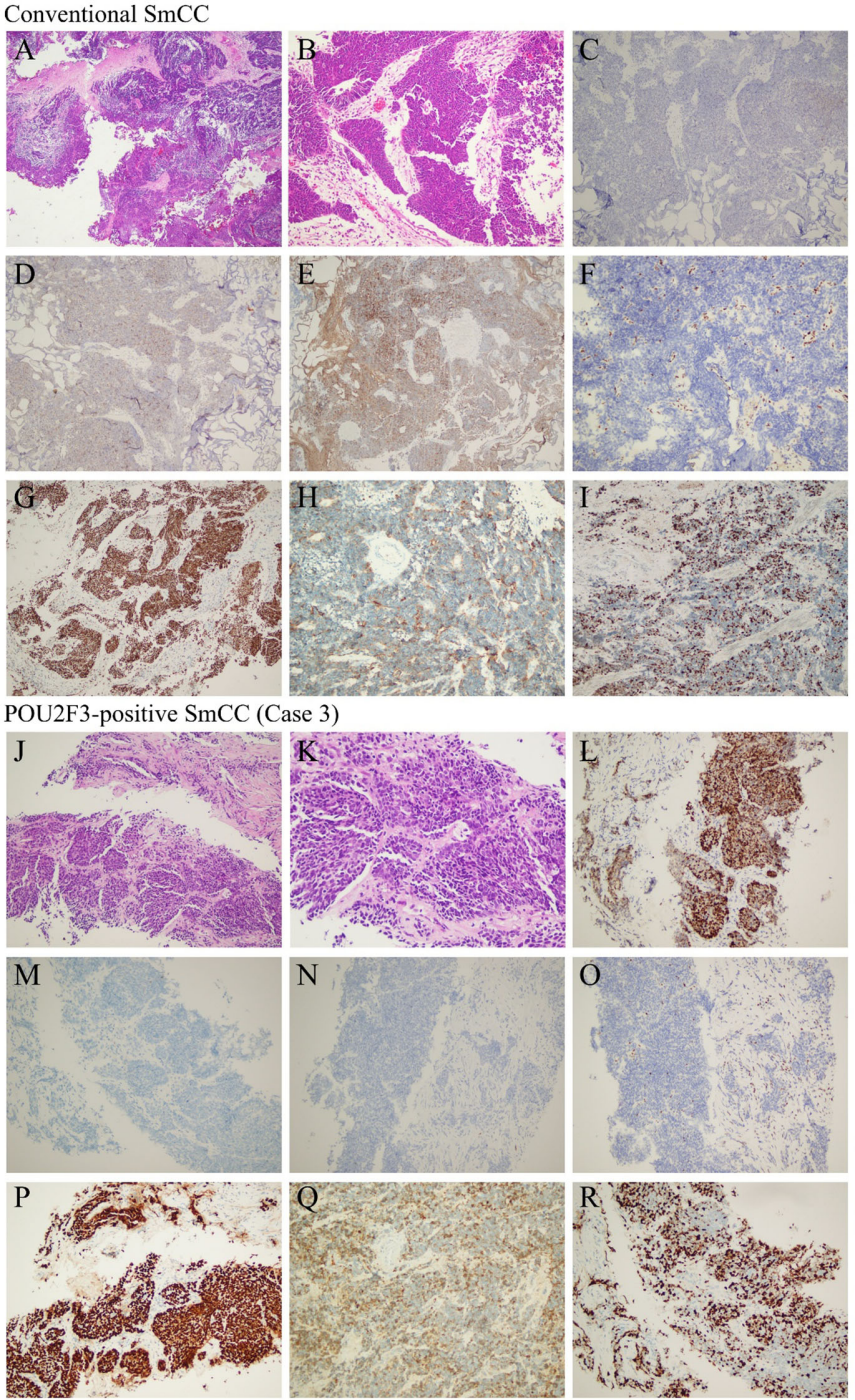
**(A–I)** Conventional small cell neuroendocrine carcinoma of the urinary tract. **(A, B)** HE staining shows tumor cells arranged in nests and sheets. **(C)** Loss of POU2F3 expression. **(D)** The neuroendocrine marker chromogranin A (CgA) shows diffuse cytoplasmic positivity. **(E)** The neuroendocrine marker synaptophysin (Syn) shows diffuse strong positivity. **(F)** Loss of RB1 expression. **(G)**. Aberrant p53 expression. **(H)** The epithelial marker AE1/AE3 shows paranuclear dot-like positivity. **(I)** High Ki-67 proliferation index. **(J–R)** POU2F3-positive small cell neuroendocrine carcinoma of the urinary tract. **(J, K)** HE staining shows tumor morphology similar to conventional small cell carcinoma. **(L)** Diffuse strong nuclear expression of POU2F3. **(M)** Loss of CgA expression. **(N)** Loss of Syn expression. **(O)** Loss of RB1 expression. (P) Aberrant p53 expression. **(Q)** AE1/AE3 shows paranuclear dot-like positivity, supporting epithelial differentiation. **(R)** High Ki-67 proliferation index.

Conventional SmCC case served as control (**Figures 1A–I**), whereas **Figures 1J–R** showed representative findings from POU2F3-positive SmCC (Case 3). HE staining demonstrated typical small cell morphology in both groups (**Figures 1A, B**, **J, K**). In the POU2F3-positive cases, POU2F3 showed diffuse strong nuclear expression in >70% of tumor cells (**Figure 1L**), while conventional SmCC cases were consistently negative for POU2F3 (**Figure 1C**). Neuroendocrine markers, including chromogranin A and synaptophysin, showed diffuse positivity in conventional SmCC (**Figures 1D, E**), but were negative in POU2F3-positive cases (**Figures 1M, N**). Loss of RB1 expression was identified in four cases (**Figure 1O**), and RB1 staining in conventional SmCC also showed loss of expression (**Figure 1F**). Aberrant p53 expression was observed in all cases, including diffuse overexpression, complete absence (null pattern), and cytoplasmic staining (**Figure 1P**). The epithelial marker AE1/AE3 showed paranuclear dot-like positivity in POU2F3-positive cases (**Figure 1Q**). Ki-67 index was high (>80%) in conventional SmCC (**Figure 1I**). Key immunohistochemical findings for each case are summarized in **Table 1**.

### Molecular findings

NGS analysis in one case revealed a TP53 missense mutation (p.V225I, exon 7).

### Treatment and follow-up

All patients received treatment, including surgery and/or chemotherapy. During follow-up, three patients developed distant metastases.

## Discussion

Primary neuroendocrine tumors of the urinary tract are rare, among which SmCC is the most common subtype. SmCC is often associated with high-grade urothelial carcinoma, whereas pure SmCC is uncommon. Morphologically, it closely resembles its pulmonary and gastrointestinal counterparts, showing sheets or nests of small round cells with scant cytoplasm, a high nuclear-to-cytoplasmic ratio, finely granular “salt-and-pepper” chromatin, inconspicuous nucleoli, and prominent nuclear molding, frequently accompanied by coagulative necrosis and the Azzopardi effect. Clinically, it is highly aggressive, with early metastasis, initial chemosensitivity, and frequent relapse. Diagnosis traditionally relies on morphology combined with epithelial and neuroendocrine markers. However, some typical cases lack or show reduced neuroendocrine marker expression, increasing diagnostic difficulty.

POU2F3, initially identified in SCLC, defines the SCLC-P subtype and is mutually exclusive with ASCL1 and NEUROD1 expression (7). This has prompted investigation of POU2F3 in extrapulmonary SmCC. Cai et al. reported that approximately one-third of bladder SmCC cases were POU2F3-positive, retaining typical morphology but showing variable neuroendocrine marker expression, with CgA frequently negative, suggesting limited sensitivity. Interestingly, POU2F3-positive cases showed a trend toward better survival (6). These findings support the potential utility of POU2F3 in recognizing this subset of urinary tract SmCC in clinical practice. Interestingly, the relatively high proportion of morphologically pure SmCC in our cohort differs from the conventional understanding that urinary tract SmCC is often associated with a conventional urothelial carcinoma component. Similar findings have been reported in previous studies of POU2F3-positive SmCC of the bladder (6). However, given the limited number of cases, whether this observation is associated with characteristic features of POU2F3-positive tumors remains to be clarified.

The histogenesis of POU2F3-positive SmCC remains unclear but may involve lineage plasticity. In the absence of native tuft cells in the urothelium, tumors may arise from tuft cell-like progenitors or through transdifferentiation of urothelial cells following TP53 and RB1 inactivation (9). The aberrant p53 expression and frequent RB1 loss observed in our cohort, which were also seen in conventional SmCC, may reflect shared molecular alterations in SmCC.

From a diagnostic perspective, the application of POU2F3 may help reduce the risk of misdiagnosis. Although the present cases exhibited typical morphological features of SmCC, conventional neuroendocrine markers were negative. In the absence of POU2F3 staining, these tumors could be easily misdiagnosed as poorly differentiated urothelial carcinoma or basaloid squamous cell carcinoma. Diffuse strong nuclear expression of POU2F3 may help exclude conventional epithelial malignancies and support classification as a neuroendocrine-low or neuroendocrine-silent phenotype of SmCC.

Given the lack of standardized screening strategies, POU2F3 immunostaining should be considered in tumors with small cell morphology but lacking neuroendocrine markers, especially in small biopsies or metastatic lesions. In terms of treatment, platinum-based chemotherapy remains the therapeutic backbone for small cell carcinoma of the bladder, largely extrapolated from treatment paradigms established in SCLC. In localized disease, neoadjuvant platinum-based chemotherapy followed by definitive local therapy is associated with superior survival compared with surgery alone and is currently the most supported treatment strategy (4, 10). In advanced or metastatic disease, platinum-etoposide remains the most commonly used first-line regimen, with carboplatin frequently substituted for cisplatin in less fit patients. Bladder-preserving chemoradiation may be considered in selected patients, although current evidence remains less robust than that for neoadjuvant chemotherapy followed by surgery (4, 10). At present, there is no direct clinical evidence supporting POU2F3-guided treatment selection.

This immunophenotypic profile may indicate biological heterogeneity. However, molecular data in urinary tract SmCC remain limited. Evidence from SCLC suggests that POU2F3-expressing tumors may have distinct therapeutic vulnerabilities (11). However, the relevance of these observations to urinary tract SmCC remains uncertain. The clinical relevance of POU2F3 in therapeutic stratification warrants further investigation.

This study has several limitations. First, its retrospective design and small sample size limit statistical power and preclude definitive conclusions. Second, because case selection was restricted to POU2F3-positive tumors, selection bias is unavoidable, and no conclusions can be drawn regarding the prevalence, sensitivity, or specificity of POU2F3 expression in urinary tract SmCC. Third, molecular characterization was available in only one case, limiting broader interpretation of the molecular features of this tumor group. Fourth, the control cohort was small and limited to conventional bladder SmCC. It was included primarily as an immunophenotypic reference rather than a formal comparative group, which limits the strength of comparison. Finally, clinical follow-up was limited, which restricts assessment of long-term outcomes and prognostic significance.

In summary, POU2F3-positive urinary tract SmCC may represent a diagnostically relevant subset with potentially different biological features. Recognition of this pattern may improve diagnostic accuracy and provide a basis for further investigation of its biological and clinical significance.

## Data Availability

The original contributions presented in the study are included in the article/supplementary material. Further inquiries can be directed to the corresponding authors.
